# Selection Criteria for Improving Fertility in Spanish Goat Breeds: Estimation of Genetic Parameters and Designing Selection Indices for Optimal Genetic Responses

**DOI:** 10.3390/ani11020409

**Published:** 2021-02-05

**Authors:** Chiraz Ziadi, Eva Muñoz-Mejías, Manuel Sánchez, María Dolores López, Olga González-Casquet, Antonio Molina

**Affiliations:** 1 Departamento de Genetica, Universidad de Cordoba, Campus de Rabanales, Ctra, Madrid–Cadiz, km 396, 14071 Cordoba, Spain; ge1moala@uco.es; 2 Departamento de Patologia Animal, Produccion Animal, Bromatologia y Tecnologia de los Alimentos, Universidad de Las Palmas de Gran Canaria, Campus Universitario Cardones de Arucas, 35413 Arucas, Spain; gerente@gescansl.com; 3 Departamento de Produccion Animal, Universidad de Cordoba, Campus de Rabanales, Ctra, Madrid–Cadiz, km 396, 14071 Cordoba, Spain; pa1sarom@uco.es; 4 Edificio de Produccion Animal, ACRIFLOR, Campus de Rabanales, Ctra, Madrid–Cadiz, km 396, 14071 Cordoba, Spain; mdoloreslopez@acriflor.org; 5 Asociacion de Criadores de la Raza Caprina Payoya, C/Arco, 23, 11680 Algodonales, Spain; payoya@payoya.com

**Keywords:** female fertility, genetic parameters, selection index, dairy goat

## Abstract

**Simple Summary:**

Up to now, the genetic evaluation of Spanish dairy goats has been based only on milk production traits. However, fertility is also an important economic trait for goats and is vital for maintaining the profitability of dairy farms, and the estimation of its genetic parameters and selection responses is, therefore, crucial for the genetic improvement of dairy goats. In this study, we estimated the genetic parameters of various fertility traits in the Florida and Payoya goat breeds, which are prototype breeds exploited under different production systems in Spain. Next, we assessed selection indices to determine the most suitable one to produce the highest number of expected selection responses. The overall interval between kiddings gives the highest genetic response but cannot be recommended as early selection criteria because it is expressed late in the female’s life, especially in the Florida breed, in which a high early culling is carried out in females with low fertility. Nevertheless, an index including the age at first kidding and the interval between the first and second kiddings can be used as a precocious selection criterion in both breeds. The results from this study can be used as a basis for the future genetic improvement of fertility traits.

**Abstract:**

The aim of this study was to estimate genetic parameters for several female fertility criteria and to choose the most suitable selection index in Spanish Florida and Payoya goat breeds. In this study, we analyzed as fertility traits, the age at first kidding (AgFiKid), and the interval between the first and second kiddings (Int12Kid), between the second, third, and remaining kiddings (Int3toKid), and between all kiddings (IntAllKid) in 51,123 and 22,049 Florida and Payoya females, respectively. Genetic parameters were estimated by fitting animal models using restricted maximum likelihood (REML) methodology. We proposed six selection indices to compare the genetic responses for all traits included, based on a new selection index theory. The heritability and repeatability estimates of the traits were low, as expected. The genetic correlations among fertility traits covered a wide range of values from 0.07 (AgFiKid-Int12Kid) to 0.71 (Int3toKid-IntAllKid) in Florida and from −0.02 (AgFiKid-Int12Kid) to 0.82 (Int3toKid-IntAllKid) in Payoya. Overall, the results of this study indicate that IntAllKid gives the highest genetic responses in both breeds but is expressed late in a female’s life. However, AgFiKid and Int12Kid could be recommended as early selection criteria for female fertility in both breeds.

## 1. Introduction

Spanish dairy goat breeds are farmed under different production systems varying from extensive, semi-extensive, and semi-intensive to intensive systems, depending on their productive aptitude and performance. The Florida and Payoya breeds are among the autochthonous dairy goats with the widest range of geographic distribution and production systems. The Florida goat can be found in different countries in the world and several regions of Spain (Extremadura, Castilla-la-Mancha, etc.), but especially in Andalusia, where it is raised under semi-extensive to semi-intensive production systems, while the Payoya breed is present only in Spain in the region of Andalusia, under semi-extensive to extensive production systems.

Nowadays, the genetic evaluation of Spanish dairy goats is based mainly on milk production traits (milk, protein, and fat yields, and protein and fat percentages), and studies have focused on estimating the genetic parameters of these traits [1,2,3]. However, other economically important traits, such as fertility, have not been studied and are not taken into consideration in the current genetic program: In fact, up to now, no estimations of genetic parameters have been made available for traits related to fertility in Spanish dairy goats.

Most studies of the genetic and environmental association between milk production and reproductive traits in various species showed an unfavorable relationship between them not only in dairy cattle [4,5] and in dairy sheep [6] but also in dairy goats [7]. Ignoring the genetic and phenotypic correlations between traits in selection programs could lead to undesirable outcomes in the correlated response of certain traits. To overcome this problem, selection indices have been developed to improve several traits related to global economic profitability simultaneously. The economic selection index implemented by Hazel (1943) [8] has been commonly used in a wide range of livestock species: meat goat [9], dairy goat [10,11], dairy cattle [12], beef cattle [13], and meat sheep [14]. Recently, this classic methodology of selection indices has been modified to overcome its limitations, to allow direct work with genetic values [15,16]. In addition to genetic values, their reliabilities, and all genetic relationships, the knowledge of traits’ economic weights is necessary. Economic weight is the value of one unit superiority of a trait when all other traits in the aggregate genotype remain constant [8]. Economic weights in goat have been derived for many traits in dairy goat: dairy, functional, and fattening traits ([17,18]).

Thus, the main objectives of the present study were to estimate the genetic parameters of fertility traits for the first time in Spanish dairy goats, using Florida and Payoya breeds as models, and to find the best selection index for the desired expected genetic response.

## 2. Materials and Methods

### 2.1. Phenotypic Data and Pedigree

In this study, we used reproductive records and pedigree information from ACRIFLOR (National Association of Florida Goat Breeders) and ACAPA (Association of Payoya Goat Breeders), which contained information on age at first kidding and the different intervals between the kiddings of Florida and Payoya females, respectively. To estimate the genetic parameters, herds with few records or scarce genetic connections were discarded. The genetic links were due to artificial insemination sires. Artificial insemination (AI) has been implemented in the Florida and Payoya breeds since 2005 within the selection scheme carried out by ACRIFLOR and ACAPA, respectively. In the last years, approximately 1808 AI with 5 bucks (from a set of 25 semen donors) and 600 AI with 3−4 bucks (from a set of 8 semen donors) were realized in Florida and Payoya, respectively. Connectedness was estimated by calculating the coefficient of similarity between herds (SG), which is the number of daughters of a connector male shared between two herds in relation to the total number of animals that have been evaluated in those two herds, establishing an SG equal to 5% as a limit for a herd to enter in the genetic evaluation process. Animals from the Payoya breed are reared under semi-extensive to extensive production systems, whereas the Florida breed presents a higher level of intensification. The following traits were considered in this study as measures of a goat’s fertility: the age at first kidding (AgFiKid), the interval between first and second kiddings (Int12Kid), the interval between second, third, and remaining kiddings (Int3toKid), and the interval between all kiddings (IntAllKid). All traits were expressed in months. After data editing, the final data set contained information on 130,849 and 67,478 reproductive records from 51,123 and 22,049 Florida and Payoya females, respectively. In total, the pedigree included 56,305 animals for Florida and 26,392 for Payoya.

### 2.2. Statistical Analysis

#### 2.2.1. Genetic Parameters of Fertility Traits

The following model was fitted to estimate the genetic parameters for all the fertility traits for Florida and Payoya breeds:

y=µ+age+Xb+Zu+Wpe+e

where y is the vector of observations of each trait, µ is the overall mean, age is a covariate representing the age at first kidding used in the model for Int12Kid or the age at kidding for the model for Int3toKid and IntAllKid; b is the vector of fixed effects including the interaction herd-year-season of kidding, in which the season of kidding was coded as 1 if a goat gave birth in the period June through September and was otherwise coded as 2, lactation length (4 levels: short, medium, long, and very long) and herd productive level (3 levels: low, medium, and high); u is the random additive genetic effect, pe is the random permanent environmental effect of the female, and e is the random residual effect. X, Z, and W are incidence matrices relating observations to fixed, random additive genetic and random female permanent environmental, respectively. The non-genetic effects in the model varied depending on the individual trait: neither the age at first kidding nor the interval between first and second kiddings had the lactation length, the herd production level, or the permanent environmental effects. The Florida and Payoya breeds were analyzed independently. Covariance components for all the traits were estimated with an animal model, using AIREMLF90 software from the BLUPF90 family of programs [19], applying a restricted maximum likelihood (REML) approach.

#### 2.2.2. Expected Genetic Responses

The genetic responses using different selection objectives/criteria were computed and compared in the Florida and Payoya breeds according to the classic selection index theory [20] reformulated by Gutiérrez et al. (2014) [15] for the use of genetic parameters rather than performances. Two selection objectives were designed (the IntAllKid, and the IntAllKid combined with the AgFiKid), and six groups of indices were constructed to combine the different fertility traits as selection criteria. In the selection indices with the second selection objective, the desired economic weight in the vector **p′** for IntAllKid was five times greater than that of AgFiKid. Weights in vector **f′** to be used for weighting the expected breeding values (EBVs) on **v** were calculated by **f′** = **p′C′G^−1^**, where **C′** is the covariance matrix between the objectives in vector **u** and the EBV used as criteria in vector **v**, and **G^−1^** is the inverse of the (co)variance matrix for the selection criteria **v**. Matrices **C′** and **G** were obtained from the genetic parameters by assuming all the additive genetic variances to be standardized $\;\left(\sigma_{u1}^{2}=\sigma_{u2}^{2}=\sigma_{u3}^{2}\cdots =\sigma_{\mathrm{uk}}^{2}=1\right)$, where $\sigma_{\mathrm{uk}}^{2}\;$ is the additive genetic variance of trait k: as a result, all of them were on the same genetic scale. When considering the different objectives and/or criteria, the coefficients in **f′** varied, and matrices **C** and **G** also changed. When objective and criteria are the same traits, the (co)variance matrix between the objectives and the criteria **C** becomes a genetic additive (co)variance matrix, in which the diagonals are equal to one [15]. Off-diagonal elements are the additive genetic correlations between objectives and criteria, given that $r_{\mathrm{ukul}}=\frac{\sigma_{\mathrm{ukul}}}{\sqrt{\sigma_{\mathrm{uk}}^{2}\sigma_{\mathrm{ul}}^{2}}},\;$ where $r_{\mathrm{ukul}}\;$ is the additive genetic correlation between traits k and l and $\sigma_{\mathrm{uk}}^{2}=1$ for any trait, thus becoming $\sigma_{\mathrm{ukul}}=r_{\mathrm{ukul}}\;$ and **C**′:

$$C^{\prime }=\mathrm{Var}\left(u\right)=\left[\begin{array}{ccccc} \sigma_{u1}^{2} & \sigma_{u1u2} & \sigma_{u1u3} & \ldots & \sigma_{u1\mathrm{um}}\\ \sigma_{u2u1} & \sigma_{u2}^{2} & \sigma_{u2u3} & \ldots & \sigma_{u2\mathrm{um}}\\ \ldots & \ldots & \ldots & \ldots & \ldots \\ \ldots & \ldots & \ldots & \ldots & \ldots \\ \sigma_{\mathrm{umu}1} & \sigma_{\mathrm{umu}2} & \sigma_{\mathrm{umu}3} & \ldots & \sigma_{\mathrm{um}}^{2} \end{array}\right]=\left[\begin{array}{ccccc} 1 & r_{u1u2} & r_{u1u3} & \ldots & r_{u1\mathrm{um}}\\ r_{u2u1} & 1 & r_{u2u3} & \ldots & r_{u2\mathrm{um}}\\ \ldots & \ldots & \ldots & \ldots & \ldots \\ \ldots & \ldots & \ldots & \ldots & \ldots \\ r_{\mathrm{umu}1} & r_{\mathrm{umu}2} & r_{\mathrm{umu}3} & \ldots & 1 \end{array}\right]$$

As **G** and **C′** are directly dependent on the genetic parameters, these matrices can be derived directly from genetic parameters to build the desired index. Next, the genetic responses were obtained by weighting, for each of the traits, all those responses obtained in the correlated selected traits, including the direct genetic response to itself. Thus, assuming the EBVs are known and applying the assumption given above about all the additive genetic variances being one, the direct genetic response would be the selection intensity (i) reduced by the accuracy of the EBV. The correlated response would be the additive genetic correlation times the selection intensity reduced by the accuracy of the EBV. Gathering this information into a matrix expression, the cumulated genetic responses will be obtained by:

$$t=b^{\prime }\mathrm{Ti}=b^{\prime }\left[\begin{array}{ccccc} h1 & h1r_{u1u2} & h1r_{u1u3} & \ldots & h1r_{u1\mathrm{uk}}\\ h2r_{u2u1} & h2 & h2r_{u2u3} & \ldots & h2r_{u2\mathrm{uk}}\\ \ldots & \ldots & \ldots & \ldots & \ldots \\ \ldots & \ldots & \ldots & \ldots & \ldots \\ {\mathrm{hkr}}_{\mathrm{uku}1} & {\mathrm{hkr}}_{\mathrm{uku}2} & {\mathrm{hkr}}_{\mathrm{uku}3} & \ldots & \mathrm{hk} \end{array}\right]\;i,$$

where each t**_k_** in t is the cumulated genetic response in trait k and h_k_ the squared root of the heritability of trait k. For the comparison of the expected responses, a constant selection intensity of one was assumed, leading to comparable relative results.

## 3. Results and Discussion

This study estimated for the first time the genetic parameters related to female fertility traits in Florida and Payoya dairy goat breeds (AgFiKid, Int12Kid, Int3toKid, and IntAllKid), and expected to find the genetic responses by combining these traits in selection indices as a step towards their incorporation into the routine genetic evaluation.

### 3.1. Phenotypic Parameters of Fertility Traits

The basic descriptive statistics for Florida and Payoya female fertility traits are presented in Table 1. The AgFiKid ranged between 12 and 24.6 months in Florida and 12 and 33 months in Payoya, with an average of 15.98 months ± 2.85 and 18.93 months ± 4.83 for Florida and Payoya, respectively, and was within the range of the values reported by other authors in US dairy goats [21,22] and Mexican Saanen goat [23], higher than those observed in Polish and Norwegian [24] and Brazilian dairy goat [25], and lower than that for the Toggenburg breed [26]. The more intensive production system (Florida) reduced AgFiKid by 88.5 days in comparison with the extensive system (Payoya). The same finding was reported between intensive (283.83 days ± 31.16) and semi-intensive (370.26 days ± 25.48) Black Bengal goats [27].

The values for Int12Kid, Int3toKid, and IntAllKid were similar in both breeds and ranged between 5.7and 17.1 months in Florida and 6.3 and 16.7 months in Payoya. Their averages varied from 10.78 months ± 1.74 (for Int12Kid in Payoya) to 11.46 months ± 1.72 (for Int3toKid in Payoya) and were lower than the ones mentioned in US dairy goats [7,19,20], but higher than the values obtained in Toggenburg [26] and Brazilian goats [25]. The coefficients of variation oscillated between 16.8% and 18.2% (for Int3toKid and Int12Kid, respectively) in Florida and 15.0 and 25.5% (for Int3toKid and AgFiKid, respectively) in Payoya. The values were, in general, similar between both breeds, except for AgFiKid, which was 30% higher in Payoya than in Florida, and lower than the 30% and 22% values reported by García-Peniche et al. (2012) [21] for AgFiKid and IntAllKid, respectively.

### 3.2. Genetic Parameters

The range of solutions for the fixed effects: herd–year–season of kidding, lactation length, and herd production level affecting fertility traits are shown in Table 2. It should be noted that all these effects, with their different levels, had a significant effect on fertility parameters, to greater or lesser degrees. This illustrated how increasing herd fertility can be achieved through environmental improvement (reproduction management, health care, feeding, etc.). The herd–year–season of kiddings’ effect had the highest levels, the widest range of values, and the most significant effect.

Estimates of the components of variance (additive genetic, permanent environmental, and residual), heritabilities (h^2^), and repeatabilities (*t*) for the evaluated traits are shown in Table 3. The estimates of the additive genetic and residual variances for AgFiKid in Payoya were about four times greater than those observed in Florida, which indicates a wider genetic and environmental variability in this breed. For the kidding interval traits, the variance estimates were quite similar for the Florida and Payoya breeds, with only a slight difference in the additive genetic variance for Int12Kid and the residual variance for Int3toKid and IntAllKid. As expected for reproductive traits, the estimates of heritabilities presented a low magnitude and varied from 0.01 ± 0.004 for Int3toKid to 0.098 ± 0.01 for AgFiKid in the Florida breed and from 0.021 ± 0.004 for IntAllKid to 0.112 ± 0.02 for AgFiKid in Payoya. The estimated h^2^ values for AgFiKid were lower than the estimates in other dairy goat breeds (0.31 ± 0.09; [23]; 0.16 ± 0.04 to 0.61 ± 0.14; [21]; 0.16 ± 0.01; [22]; 0.21; [25]). The h^2^ estimates for kidding intervals in both breeds were within the range of values reported in other studies (0.015 ± 0.036 and 0.03 ± 0.007; [24]; 0.00 to 0.15 ± 0.06; [7]; 0.02 ± 0.01 to 0.08 ± 0.02; [21]; 0.09 ± 0.02; [22]; 0.06; [25]). Other authors [28] in a meta-analysis of several studies on dairy goats have reported h^2^ values of 0.17 ± 0.012 for AgFiKid, 0.002 ± 0.018 for the first kidding interval, and 0.09 ± 0.01 for IntAllKid.

The repeatability for Int3toKid and IntAllKid showed very low estimated values, of the same magnitude as their corresponding heritabilities in both breeds (0.033−0.03 for Int3toKid and 0.023−0.022 for IntAllKid in Florida and Payoya, respectively), and similar to the values obtained by García-Peniche et al. (2012) [21], dos Santos et al. (2015) [25], and the weighted average estimated by Jembere et al. (2017) [28] from six studies. Due to their low heritability and repeatability, kidding interval traits depend mostly on environmental conditions and could be improved by management practices in addition to genetic selection. Moreover, the decision on culling females should be taken based on more than one record.

The estimates of genetic correlations among fertility traits are shown in Table 4. They were positive in all cases, except the value observed for the r_g_ between AgFiKid and Int12Kid in Payoya, and covered a wide range of values. The genetic correlations between AgFiKid and kidding interval traits ranged from 0.07 (AgFiKid-Int12Kid) to 0.19 (AgFiKid-Int3toKid) in Florida and from −0.02 (AgFiKid-Int12Kid) to 0.07 (AgFiKid-Int3toKid) in Payoya. Estimated r_g_ among kidding intervals oscillated between 0.24 (Int12Kid-Int3toKid) and 0.71 (Int3toKid-IntAllKid) in Florida, and between 0.12 (Int12Kid-Int3toKid) and 0.82 (Int3toKid-IntAllKid) in the Payoya breed.

Correlations between age at first kidding and kidding intervals in other studies were reported to be negative (−0.71 ± 0.27 in Anglo-Nubian goats [29]; −0.43 ± 0.11 in Arsi-Bale goats [30]) or positive (0.64 ± 0.01 in Saanen goats [29]. These values indicate that the magnitude and the sign of direct and indirect responses for selection for these traits will depend heavily on the choice of the selection criteria. The use of AgFiKid as a selection criterion was demonstrated to increase female productive life at 72 months by 2.77 days per generation, while selecting for IntAllKid decreased female productive life at 72 months by 8.28 days per generation [22].

In general, however, there are few estimates available in the literature of the genetic correlations among fertility traits in dairy goats.

### 3.3. Expected Genetic Responses

The selection indices in this study were computed based on genetic parameters rather than phenotypic performances according to the methodology developed by other authors [15]. This approach has the advantage of using accurate values adjusted for the environmental effects. In addition, it allows a natural connection between the net merit of an animal’s genotype and its relationship to profitability [16]. Many authors working with selection indices (in dairy cattle [31], in beef cattle [13], in meat sheep [32], and dairy goats [11]) have demonstrated that the selection index is extremely accurate for animal selection because it takes into account the genetic relationship between traits and their economic weights.

The expected genetic responses for the different fertility indices in Florida and Payoya breeds are presented in Table 5. The negative responses obtained indicated a lower age at the first kidding, shorter time intervals, and then higher fertility, and the highest values were observed in the Florida breed under all selection indices. This was due to the fact that estimated genetic parameters (h^2^ and r_g_) of fertility traits were higher in most cases in this breed. In general, the selection indices with combined criteria give greater selection responses than when a single criterion was used, and index 5 and the full index (index 6) presented the highest genetic responses. Moreover, it should be noted that the responses were higher when IntAllKid was included in the selection objective/criteria. In the selection indices with IntAllKid as a selection objective, the maximum expected genetic response was achieved in indices 3, 5, and 6 in Florida (−0.0106 months per generation) and Payoya (−0.0088 months per generation). In the case of a combined selection objective (IntAllKid and AgFiKid), the maximum response was obtained in indices 5 and 6 in Florida (−0.0710 months per generation) and Payoya (−0.0498 months per generation).

The expected responses varied in magnitude depending on the defined selection objectives, the traits included as criteria in the selection indices, and their genetic parameters (heritability and genetic correlations). The best global responses were achieved when both IntAllKid and AgFiKid were combined in the selection objective and when the indices included IntAllKid as a selection criterion. This can be explained by the fact that IntAllKid provides the maximum amount of information on fertility traits, which increases selection accuracy and optimizes genetic response. However, in practice, this trait is expressed later in the female’s life, and direct selection for this trait will reduce this gain in accuracy by delaying the generation interval. Poor fertility is one of the most common reasons for culling females (only 52.7% of Florida females have more than six parities, compared with 78.6% in the Payoya breed), even at an early age, especially in the Florida breed, where 24.7% of females are eliminated between the first and second parities (14.8% in Payoya). Using precocious selection criteria, such as AgFiKid and Int12Kid, separately (indices 1 and 2), will generate a very slow genetic response, especially for the Payoya breed. Nevertheless, in both breeds, it could be possible to select females based on these two criteria together under a combined selection goal (AgFiKid and Int12Kid: index 4, Table 5). Moreover, further work is required in this area, especially on exploring new selection criteria that are positively correlated with IntAllKid, and which are expressed early in the animal’s life and produce the desired genetic response.

## 4. Conclusions

Fertility traits are very important for any breeding farm, regardless of the productive objectives. Herd–year–season, lactation length, and herd production level are the key non-genetic factors affecting fertility traits, which suggest that increasing goat fertility can be achieved by improving these factors. As expected for these types of traits, the heritability estimates were of a low magnitude, which implies that direct selection for most fertility traits would lead to slow direct and indirect genetic responses. In the case of the Payoya breed, the heritability estimates were lower than those in the Florida breed, which is most likely due to its more extensive production system. The interval between all kiddings could be the best selection criteria for improving fertility, but this trait is expressed late in female’s life. The use of the age at first kidding and the interval between first and second kiddings as selection criteria is recommended as a precocious criterion in both breeds.

Finally, despite the limited heritability estimates obtained for fertility traits in this study, their magnitude is high enough to ensure the genetic improvement of these traits. Modifying fertility aspects in these two breeds could be achieved by selection for one of these traits, but given the low genetic response obtained with current precocious selection criteria (AgFiKid and Int12Kid), further research is needed into other fertility criteria that are expressed early in the animal’s life.

## Figures and Tables

**Table 1:** Basic descriptive statistics for fertility traits in Florida and Payoya breeds.

Item	N° of Records		Mean ± SD		Min		Max		CV (%)	
Traits ^1^	Florida	Payoya	Florida	Payoya	Florida	Payoya	Florida	Payoya	Florida	Payoya
AgFiKid	40.652	13.909	15.98 ± 2.85	18.93 ± 4.83	12	12	24.6	33.0	17.83	25.52
Int12Kid	24.106	9.108	10.87 ± 1.98	10.78 ± 1.74	5.7	6.3	17.1	16.5	18.23	16.15
Int3toKid	42.858	23.623	11.41 ± 1.91	11.46 ± 1.72	5.7	6.3	17.1	16.7	16.77	15.00
IntAllKid	75.315	34.006	11.22 ± 2.01	11.28 ± 1.77	5.7	6.3	17.1	16.7	17.88	15.67

^1^: all traits are expressed in months; AgFiKid: age at first kidding; Int12Kid: interval between first and second kiddings; Int3toKid: interval between second, third, and remaining kiddings; IntAllKid: interval between all kiddings; SD: standard deviation; Min: minimum; Max: maximum; CV: coefficient of variation.

**Table 2:** Solutions for fixed effects for fertility traits in Florida and Payoya breeds ^1^.

Effects	Levels	AgFiKid		Int12Kid		Int3toKid		IntAllKid	
		Florida	Payoya	Florida	Payoya	Florida	Payoya	Florida	Payoya
Herd-year-season ^2^	1 to *n*	12.36 to 24.38 (876)	12.15 to 28.09 (328)	8.29 to 15.57 (620)	7.91 to 13.75 (278)	9.63 to 16.27 (774)	9.32 to 15.39 (338)	9.39 to 16.51 (1072)	8.02 to 16.70 (389)
Lactation length ^3^	1	-	-	−1.32 (0.021)	−0.92 (0.05)	−2.97 (0.025)	−2.75 (0.027)	−2.84 (0.017)	−2.61 (0.021)
	2	-	-	0.0 -	0.0 -	−1.28 (0.016)	−1.65 (0.016)	−1.28 (0.012)	−1.61 (0.014)
	3	-	-	1.13 (0.023)	1.39 (0.07)	0.0 -	0.0 -	0.0 -	0.0 -
	4	-	-	2.86 (0.029)	2.81 (0.10)	1.69 (0.020)	1.35 (0.019)	1.73 (0.016)	1.36 (0.017)
Herd production level ^3^	1	-	-	−0.16 (0.019)	0.0 -	−0.28 (0.024)	−0.04 (0.019)	−0.13 (0.014)	−0.12 (0.014)
	2	-	-	0.0 -	0.25 (0.027)	−0.13 (0.015)	0.0 -	0.0 -	0.0 -
	3	-	-	0.18 (0.020)	0.66 (0.067)	0.0 -	0.11 (0.017)	0.13 (0.011)	0.14 (0.016)

^1^: solutions of the different levels of the fixed effects are significantly different at 99%; ^2^: number of levels for herd–year–season effect is presented between parenthesis; ^3^: standard errors of estimates are presented between parenthesis; AgFiKid: age at first kidding in months; Int12Kid: interval between first and second kiddings in months; Int3toKid: interval between second, third, and remaining kiddings in months; IntAllKid: interval between all kiddings in months.

**Table 3:** Estimates of genetic parameters of fertility traits in Florida and Payoya breeds ^1^.

Traits	AgFiKid		Int12Kid		Int3toKid		IntAllKid	
Parameters	Florida	Payoya	Florida	Payoya	Florida	Payoya	Florida	Payoya
$\sigma_{a}^{2}$	0.463 (0.05)	1.86 (0.3)	0.088 (0.014)	0.025 (0.013)	0.013 (0.005)	0.022 (0.004)	0.024 (0.005)	0.018 (0.004)
$\sigma_{\mathrm{pe}}^{2}$	-	-	-	-	0.03 (0.008)	0.0015 (0.002)	0.007 (0.006)	0.0013 (0.002)
$\sigma_{e}^{2}$	4.249 (0.05)	14.71 (0.32)	0.93 (0.015)	0.81 (0.018)	1.23 (0.01)	0.76 (0.008)	1.29 (0.008)	0.83 (0.007)
$h^{2}$	0.098 (0.01)	0.112 (0.02)	0.086 (0.013)	0.03 (0.016)	0.01 (0.004)	0.028 (0.005)	0.018 (0.004)	0.021 (0.004)
t	-	-	-	-	0.033	0.03	0.023	0.022

^1^: standard errors of estimates are presented between parenthesis; AgFiKid: age at first kidding in months; Int12Kid: interval between first and second kiddings in months; Int3toKid: interval between second, third, and remaining kiddings in months; IntAllKid: interval between all kiddings in months;$\;\sigma_{a}^{2}$: additive genetic variance;$\;\sigma_{\mathrm{pe}}^{2}$: permanent environmental variance;$\;\sigma_{e}^{2}$: residual variance; $h^{2}$: heritability; *t*: repeatability.

**Table 4:** Estimates of genetic correlations among fertility traits in Florida and Payoya breeds ^1^.

Item	Int12Kid		Int3toKid		IntAllKid	
	Florida	Payoya	Florida	Payoya	Florida	Payoya
AgFiKid	0.07	−0.02	0.19	0.07	0.11	0.05
Int12Kid	-	-	0.24	0.12	0.58	0.58
Int3toKid	-	-	-	-	0.71	0.82

^1^: all estimates of genetic correlations are significantly different from 0 at the 0.05 level; AgFiKid: age at first kidding in months; Int12Kid: interval between first and second kiddings in months; Int3toKid: interval between second, third, and remaining kiddings in months; IntAllKid: interval between all kiddings in months.

**Table 5:** Expected genetic responses for the fertility indices in Florida and Payoya breeds ^1^.

Indices	Selection Criteria	Selection Objectives			
		IntAllKid		IntAllKid, AgFiKid	
		Florida	Payoya	Florida	Payoya
1	AgFiKid	−0.0001	−0.0000	−0.0447	−0.0277
2	Int12Kid	−0.0035	−0.0029	−0.0150	−0.0061
3	IntAllKid	−0.0106	−0.0088	−0.0431	−0.0279
4	AgFiKid, Int12Kid	−0.0036	−0.0030	−0.0535	−0.0352
5	AgFiKid, IntAllKid	−0.0106	−0.0088	−0.0710	−0.0498
6	All traits	−0.0106	−0.0088	−0.0710	−0.0498

^1^: expected genetic responses for fertility traits are expressed in months per generation; AgFiKid: age at first kidding; Int12Kid: interval between first and second kiddings; IntAllKid: interval between all kiddings.

## Data Availability

Not applicable.
